# Two-Step Pseudomaximum Amplitude-Based Confidence Interval Estimation for Oscillometric Blood Pressure Measurements

**DOI:** 10.1155/2015/920206

**Published:** 2015-10-04

**Authors:** Soojeong Lee, Gwanggil Jeon, Seokhoon Kang

**Affiliations:** ^1^Department of Electronics and Computer Engineering, Hanyang University, 222 Wangsimni-ro, Seongdong-gu, Seoul 133-791, Republic of Korea; ^2^Department of Embedded Systems Engineering, Incheon National University, 119 Academy-ro, Yeonsu-gu, Incheon 406-772, Republic of Korea

## Abstract

Blood pressure (BP) is an important vital sign to determine the health of an individual. Although the estimation of average arterial blood pressure using oscillometric methods is possible, there are no established methods for obtaining confidence intervals (CIs) for systolic blood pressure (SBP) and diastolic blood pressure (DBP). In this paper, we propose a two-step pseudomaximum amplitude (TSPMA) as a novel approach to obtain improved CIs of SBP and DBP using a double bootstrap approach. The weighted median (WM) filter is employed to reduce impulsive and Gaussian noises in the step of preprocessing. Application of the proposed method provides tighter CIs and smaller standard deviation of CIs than the pseudomaximum amplitude-envelope and maximum amplitude algorithms with Student's *t*-method.

## 1. Introduction

The maximum amplitude algorithm (MAA) based on oscillometric measurement is the most widely used technique to estimate the average arterial blood pressure [1–5]. The MAA approximates the mean blood pressure as the cuff pressure (CP) at which the maximum oscillation occurs and then linearly relates the systolic blood pressure (SBP) and diastolic blood pressure (DBP) [2, 6]. The blood pressure is constantly changing because of intrinsic physiological oscillations and in response to factors such as stress, exercise, disease, and food. Thus, the SBP and DBP can shift up to 20 mmHg within a few heartbeats and have larger variations over the course of the day [7]. This phenomenon and its serious consequences on blood pressure (BP) measurement are not recognized by most physicians, and it is what makes accurate measurements of BP a difficult task [8]. The American National Standard Institute (ANSI)/Association for the Advancement of Medical Instrumental (AAMI) [9] recommends a maximum allowable system error of ±5 mmHg with standard deviation of 8 mmHg compared to a reference reading done simultaneously by at least two trained nurses. However, the actual physiological variability, which could reach up 20 mmHg, is used to be ignored [8].

Although the oscillometric blood pressure devices are popularly used to estimate the SBP and DBP, these devices provide only one estimate with no confidence interval (CI) and users are not able to distinguish the statistical variance in the estimates from the intrinsic variability exhibited by physiological processes [10]. Since there is no golden standard technique except for auscultatory method, there is no method to determine the variance in BP estimates. If the CI in the BP estimates is too wide, an alert can recommend discarding the measurements and initiating another measurement. Without the CI, it is difficult to make any meaningful decision with the BP estimates. Based on some aggregate statistics, in a home-based monitoring setting, the repeated wide CI can trigger an alarm and alert either the nurse station or the family doctor. Even though this is an important factor of blood pressure estimation, until very recently, there was no research investigating the estimation of the CI for these blood pressure measurements. Recently, Krakoff [11] proposed that the CIs were computed for SBP, DBP, pulse rate, and heart rate obtained from an Omron HEM725CIC monitor over a period of 7 days, with four measurements per patient (28 measurements per patient), which is not considered large BP measurements. Hence, the Student's *t*-distribution (ST), instead of asymptotic normal distribution, was utilized to obtain the CI of the SBP and DBP [11]. Although the asymptotic normal approximation is generally used to derive CIs, a large sample size is inevitably required to obtain such CIs. However, it is not feasible to acquire a large number of measurements for each subject using a noninvasive oscillometric blood pressure measurement device, as repeatable conditions for reproducible measurements cannot be guaranteed [12]. As a consequence, standard methods of obtaining the CI such as the one presented in [11] cannot be used for obtaining the CI in blood pressure measurements. This calls for an innovative method that can obtain the CI from a smaller sample size. In this regard, bootstrap approaches with oscillometric blood pressure measurements were presented in [12]. On the other hand, in our previous study [12], we confirmed that the CI using the bootstrap method sometimes becomes too wide or too narrow or too wide in one direction and too narrow in the other because only five measurements for each subject are used. That is, the standard variation of CIs is larger than the average of CIs for the SBP and DBP. Therefore, it is necessary to develop a method that can correct the problem of the CI obtained using a small number of measurements [13]. In this paper, we propose a two-step pseudomaximum amplitude (TSPMA) as a novel method to obtain improved CIs of SBP and DBP using a double bootstrap method [14]. The CIs based on the double bootstrap are significantly to reduce coverage rate errors obtained from single bootstrap method [15]. In particular, the TSPMAs are efficiently obtained from the pseudomaximum amplitudes (PMAs) which are large resample vectors due to the increase in the number of samples using the double bootstrap. Thus, we address the problem of CIs using the TSPMA based on the double bootstrap for the SBP and DBP. Moreover, we perform various experiments in the impulse and Gaussian noisy environments to evaluate the performance of the proposed algorithm. Summarizing our approach, this paper can be regarded as an expanded version of the previous paper [12] with the following enhancements:

- developing a method that reduces the standard deviation of the CI of PMA [12] using a small number of measurements;
- using the weighted median (WM) filter to reduce impulsive and Gaussian noises.

Extensive simulation results show that the proposed algorithm offers tighter CI and smaller CIs' standard variation than the conventional algorithms.

## 2. Methods

Indeed, it is not feasible to obtain a large sample from each subject in BP measurement due to cost reasons. Even when cost is not the core issue, experimental conditions may not provide reproducible BP measurements. In such scenarios, one may have to resort to the method of employing pseudomeasurements as introduced in [12]. In this study, pseudomeasurements are also used to obtain the CI using the double bootstrap technique. The proposed method consists of two main steps to obtain the TSPMA and pseudoenvelope (PE) so that our approach is called two-step pseudomaximum amplitude-pseudoenvelope (TSPMAE).

**Figure 1:**
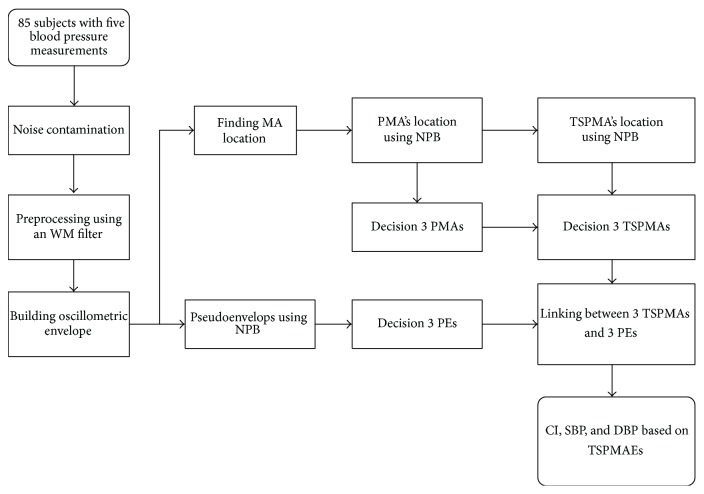
Procedure of TSPMAE based on NPB for improved confidence interval (CI) estimator.

The block diagram of the proposed approach is given in Figure 1. The upper path of the block diagram shows the first step including the PMA and TSPMA parts of the algorithm, whereas the lower path of the block diagram shows the second step regarding the PEs. These two steps are then utilized to get the CI estimate of BP. The envelopes of oscillometric BP are smoothed using the Gaussian curve fitting and separated into systolic BP and diastolic BP parts of the envelope. These envelopes are used in the lower path as shown in Figure 1. In the upper path, we obtain the maximum amplitude (MA) locations using the MAA technique. Then, the PMA locations are obtained using the nonparametric bootstrap (NPB) [12, 17]. We then also obtain the TSPMA locations reusing the PMA locations based on the NPB. In the next step, the upper, middle, and the lower PMAs and TSPMAs and the locations corresponding to those PMAs and TSPMAs are determined by using the CI technique [12].

In the lower path where the Gaussian curve fitted envelopes are adapted to get the same lengths, two sets of envelope matrices, which are systolic BP and diastolic BP parts, are constructed. The PEs are obtained for systolic BP and diastolic BP parts using the bootstrap technique. By employing the NPB for CI, the upper, middle, and lower PEs are then obtained. Following this, the results from TSPMA path are used to link the PEs and TSPMAs; then the CI estimates are obtained using the mean cuff pressure (MCP) which is computed based on the CP of the five measurements. For more details on the PMA and PEs, the interested readers are referred to [12].

In particular, the preprocessing component is to suppress a noisy signal using the WM filter [18], which is a set of *K* BP envelope valued weight 〈*W*_1_, *W*_2_,…, *W*_*K*_〉 and the observation peak vector *X* = [*X*_1_, *X*_2_,…, *X*_*K*_]^*T*^. Thus, the WM filter's output is given by the use of the median operator such that

(1) $$\begin{array}{c} \hat{\beta }=\text{median}\left(\left|W_{1}\right|◊\mathrm{sgn}\left(W_{1}\right)X_{1},\ldots ,\left|W_{K}\right|◊\mathrm{sgn}\left(W_{K}\right)X_{K}\right), \end{array}$$

where ◊ denotes the replication operator and *W*_*i*_ ∈ *R* denotes the weighted value for *i* = 1,2,…, *K*. Note that the weight signs are uncoupled from the magnitude values of BP envelope and are merged with the observation BP envelope sample as follows [19]:

(1) calculate the threshold *T*_0_ = (1/2)∑_*i*=1_^*K*^ | *W*_*i*_|;
(2) sort the signed observation sample sgn(*W*_*i*_)*X*_*i*_;
(3) sum the magnitude of the weights corresponding to the sorted “signed” samples beginning with the maximum and continuing down in order;
(4) output is the signed sample whose weight magnitude causes the sum to become ≥*T*_0_.

### 2.1. Short Review of Bootstrap

The fundamental concept of the bootstrap technique [17] is to provide a large number of independent bootstrap BP estimates by resampling the original BP estimate *X* = (*x*_1_, *x*_2_,…, *x*_*n*_) of *n* measurements at random from an unknown probability distribution *F*. Bootstrap resamples *X*_1_^*^( = *x*_1_^*^, *x*_2_^*^,…, *x*_n_^*^),…, *X*_B_^*^( = *x*_1_^*^, *x*_2_^*^,…, *x*_n_^*^) are acquired by sampling *n* time randomly drawn with replacement from the original sample *X* with elements occurring zero, once, or multiple times, where *n* denotes an original sample size and *B* denotes a number of resamples. Based on the approach done by Efron and Tibshirani [17], we use *B* = 1000 for CIs. However, this number relies on the particular application. Specifically, as we have only five measurements for each subject, the number of all the possible bootstrap resamples is given as (2*n* − 1)!/[*n*!(*n* − 1)!]. This indicates that the number of bootstrap resamples achieves stability [20] as *B* approaches 126.

In this paper, we determine the CIs of oscillometric BP measurements using the nonparametric bootstrap [17, 21]. Specifically, the CI ${\hat{\theta }}_{\alpha }^{*}$ with nonparametric bootstrap (NPB) represents the 100 · *α*th percentile of *B* bootstrap replications ${\hat{\theta }}_{\left(1\right)}^{*},{\hat{\theta }}_{\left(2\right)}^{*},\ldots ,{\hat{\theta }}_{\left(B\right)}^{*}$. Percentile limit ${\hat{\theta }}_{l}$, ${\hat{\theta }}_{u}$ of intended range 1 − 2 · *α* is simply obtained such that

(2) $$\begin{array}{c} \left({\hat{\theta }}_{l},{\hat{\theta }}_{u}\right)=\left({\hat{\theta }}_{\left(\alpha \left(B+1\right)\right)}^{*},{\hat{\theta }}_{\left(1-\alpha \right)\left(B+1\right)}^{*}\right), \end{array}$$

where *l* and *u* are the lower and upper bounds of the CI and *α* is set to 0.05.

### 2.2. Review of PMA Using NPB [12]

**Figure 2:**
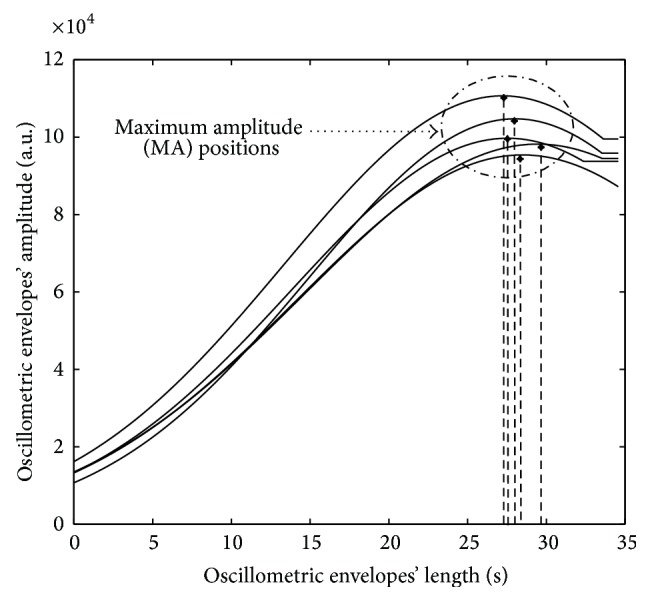
The MA's positions from a subject with five measurements [12].

In this section, we briefly represent the methodology to determine the upper and lower bounds on the CI for MA and the length position of the MA on the oscillometric BP envelope. The NPB method is used to determine the mean of the CI and the range for the SBP and DBP [12]. Firstly, we obtain the MAs and the length of occurrence of the MAs from all the five measurements per subject, respectively, as shown in Figure 2. These raw parameters of the MAs are utilized to find the final PMAs which are then used to obtain the TSPMA using the NPB method [12] as a special case of the double bootstrap method [14].

**Figure 3:**
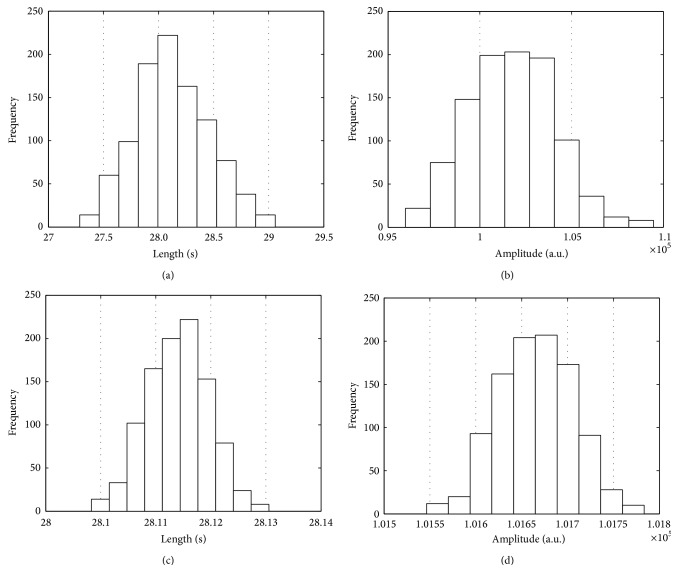
Histograms of the PMA and TSPMA using NPB for one subject. (a) The length positions of the amplitude of the PMA, (b) the amplitude of the PMA, (c) the length positions of the amplitude of the TSPMA, and (d) the amplitude of the TSPMA.

Suppose that *X* = {*x*_1_,…, *x*_5_} denote the set of five length positions of the MAs and *Y* = {*y*_1_,…, *y*_5_} denote the set of the corresponding five MAs. Based on the NPB on these two sets, we create *B* of resamples, *X*_j_^*^, *Y*_j_^*^, *j* = 1,…, *B*, where *X*_j_^*^ = {*x*_1j_^*^,…, *x*_5j_^*^} and *Y*_j_^*^ = {*y*_1j_^*^,…, *y*_5j_^*^}, respectively. We then compute the mean of all measurements in *X*_j_^*^ and *Y*_j_^*^ to obtain ${\hat{\mu }}_{X(j)}^{*}$ and ${\hat{\mu }}_{Y(j)}^{*}$ given by ${\hat{\mu }}_{X(j)}^{*}=(1/N)\sum_{k=1}^{N}x_{k,j}^{*}$ and ${\hat{\mu }}_{Y(j)}^{*}=(1/N)\sum_{k=1}^{N}y_{k,j}^{*}$, where *N* = 5 and *j* = 1,…, *B*. The histograms of the bootstrap estimates ${\hat{\mu }}_{X(j)}^{*}$ and ${\hat{\mu }}_{Y(j)}^{*}$ are expressed in Figures 3(a) and 3(b) which represent the length of occurrence of the maxima and PMA from all the five measurements per subject, respectively. We then sort the bootstrap estimates, ${{\hat{\mu }}^{*}}_{X(j)}$ and ${{\hat{\mu }}^{*}}_{Y(j)}$, according to ascending order. Therefore, the sorted PMAs are acquired as ${{\hat{\mu }}^{*}}_{Y(1)}\leq {{\hat{\mu }}^{*}}_{Y(2)}\leq {{\hat{\mu }}^{*}}_{Y(3)}\cdots \leq {{\hat{\mu }}^{*}}_{Y(B-1)}\leq {{\hat{\mu }}^{*}}_{Y(B)}$ and the length locations of the PMAs are acquired as ${{\hat{\mu }}^{*}}_{X(1)}\leq {{\hat{\mu }}^{*}}_{X(2)}\leq {{\hat{\mu }}^{*}}_{X(3)}\cdots \leq {{\hat{\mu }}^{*}}_{X(B-1)}\leq {{\hat{\mu }}^{*}}_{X(B)}$. Indeed, the desired 100 · (1 − *α*)% nonparametric CIs for position of PMA and the PMA are, respectively, acquired as $\left({{\hat{\mu }}^{*}}_{X(Q_{1})},{{\hat{\mu }}^{*}}_{X(Q_{2})}\right)$ and $\left({{\hat{\mu }}^{*}}_{Y(Q_{1})},{{\hat{\mu }}^{*}}_{Y(Q_{2})}\right)$, where *Q*_1_ is the quotient of *B* · *α*/2, *Q*_2_ = *B* − *Q*_1_ + 1, and *Q*_3_ = *B*/2. We therefore obtain *Q*_1_ = 25, *Q*_2_ = 976, and *Q*_3_ = 500 with *α* = 0.05 and *B* = 1000. Finally, we get the three positions of the PMA that will be used by the algorithm to estimate CIs of the SBP and DBP, respectively.

### 2.3. Proposed TSPMA Using Double Bootstrap

The main goal of the TSPMA technique based on the double bootstrap is to provide improved CIs of SBP and DBP with respect to the subject using only five measurements.

In practice, the first step is to abandon the mean as a measure of center in favor of a statistic that is more resistant to outliers [13]. The trimmed mean is the mean of only the center observations in a data set. In particular, the 25% trimmed mean ignores the smallest and largest 25% of the observations [13]. Thus, we acquire pseudomeasurements $\zeta^{*}=\{{\hat{\mu }}_{X(Q_{4})}^{*},\ldots ,{\hat{\mu }}_{X(Q_{5})}^{*}\}$ and $\eta^{*}=\{{\hat{\mu }}_{Y(Q_{4})}^{*},\ldots ,{\hat{\mu }}_{Y(Q_{5})}^{*}\}$ from (3) as vectors of bootstrap resample, respectively, where *Q*_4_ is 251( = 0.25 × *B* + 1) and *Q*_5_ is 750( = *B* − *Q*_4_ + 1). Herein, we also create a number of *B*( = 1000) of resamples *ζ*_j_^**^ = {*χ*_1j_^**^,…, *χ*_500j_^**^} and *η*_j_^**^ = {*ψ*_1j_^**^,…, *ψ*_500j_^**^} applied from the PMA's set obtained by trimmed means which are *ζ* ^*^(1 × 500) and *η* ^*^(1 × 500), using the NPB for *j* = 1 to *B*, respectively. Thus, we obtain two matrices (500 × 1000) such as *ζ*_j_^**^ and *η*_j_^**^ which are calculating the mean of all measurements, where the mean of all resample measurements is given as follows:

(3) $$\begin{array}{c} {\hat{\mu }}_{\chi (j)}^{**}=\frac{1}{B_{2}}\overset{B_{2}}{\underset{k=1}{\sum }}\chi_{kj}^{**},\\ {\hat{\mu }}_{\psi (j)}^{**}=\frac{1}{B_{2}}\overset{B_{2}}{\underset{k=1}{\sum }}\psi_{kj}^{**}, \end{array}$$

where ${\hat{\mu }}_{\chi (j)}^{**}$ denotes the TSPMA and ${\hat{\mu }}_{\psi (j)}^{**}$ denotes the length positions of TSPMA, which become vectors (1 × 1000). Also, *B*_2_( = 500) is the number of the resamples obtained from *Q*_4_ to *Q*_5_.

In the next step, we also sort the TSPMAs and the length positions of the TSPMA in increasing order. The desired 100 · (1 − *α*)% double bootstrap's CIs for position of TSPMA and the TSPMA are, respectively, given by $\left({{\hat{\mu }}^{**}}_{\chi (Q_{1})},{{\hat{\mu }}^{**}}_{\chi (Q_{2})}\right)$ and $\left({{\hat{\mu }}^{**}}_{\psi (Q_{1})},{{\hat{\mu }}^{**}}_{\psi (Q_{2})}\right)$, where *Q*_1_ is the quotient of (*B* · *α*)/2, *Q*_2_ = *B* − *Q*_1_ + 1, and *Q*_3_ = *B*/2, where *α* = 0.05 and *B* = 1000.

### 2.4. Review of PE Using NPB [12]

In order to obtain the PEs for estimating the CI of the SBP and DBP using NPB, we construct a BP measurement matrix **E** as shown in [12] composing BP envelopes for five measurements for the systolic and diastolic parts of each subject [12]. Each column of BP measurement matrix **E** denotes an BP envelope obtained from the oscillometric measurement. Particularly, all measurements are forced to be of length, either by extrapolating length if the measurement is shorter or by truncating the length if the measurement is longer. From the BP envelope matrix **E**, employing NPB method, we acquire *B* resample envelope matrices **E**_1_^*^,…, **E**_B_^*^, where *j* = 1,…, *B*( = 1000). The SBP and DBP parts of the envelope are identified utilizing the peak of the envelope. From the beginning to the peak of the BP envelope (corresponding to the decreasing cuff pressure) represents the SBP part and from the peak to the end of the BP envelope represents the DBP part. We then reorder the resampled BP envelope matrices (for systolic and diastolic parts of the envelopes) using the ascending and descending sort techniques (for SBP and DBP parts of the envelopes, resp.). It is noted that each of the sorted matrices has five columns, each corresponding to a BP measurement of length *L*. We then obtain a single BP envelope per subject as shown in [12]. For more details on the PE, the reader is referred to [12].

In the previous subsection, we obtain the value of the TSPMA utilizing the NPB approach. As the TSPMA estimates may not connect with the end (start) point of the systolic (diastolic) PEs and also in the amplitudes, it may be needful to use signal processing (padding and clipping) to ensure that the location values (both in amplitude and in length) of the TSPMA are based on the PEs. In the final step, we need to obtain the MCP to find the CI estimates of SBP and DBP. In order to estimate the SBP and DBP, systolic and diastolic ratios must be determined. The systolic and diastolic ratios used in our algorithm are 0.70 and 0.45, respectively, which were experimentally decided [1, 2]. Using these ratios, the SBP and DBP points are identified on the TSPMAE, and they are mapped back to the MCP in the SBP and DBP values in mmHg.

## 3. Results

This work was approved by the local research ethics committee, and all subjects offered informed consent prior to the BP measurement on the basis of the protocol of the institutional research ethics board. The oscillometric measurements were provided by Biosign Technologies Inc., Toronto, Ontario, Canada, for this work. The experimental BP data set was acquired from 85 healthy subjects aged from 12 to 80, out of which thirty-seven were females and forty-eight were males. Oscillometric BP measurements were obtained from each volunteer (5 set ×85 subjects = 425 total measurements based on a wrist worn UFIT TEN-10 blood pressure device) (Biosign Technologies Inc., Toronto, Ontario, Canada) in accordance with the recommendations of the ANSI/AAMI SP 10 standard [9]. In particular, the two nurse reference readings at the same time are averaged to supply one SBP and one DBP reading. Nurse reference reading of SBP ranged from 78 to 147 mmHg and DBP ranged from 42 to 99 mmHg across total subjects [22]. Note that our procedure of BP measurements consists of an oscillometric blood pressure recoding, followed by readings of SBP and DBP with the help of two trained nurses after a one-minute pause. This was then followed by another one-minute break. The procedure was repeated again four more times to build the recoding of five measurements [22]. Thus, we only acquired the five measurements for each subject because it is not practically possible to obtain a large number of measurements [12].

In order to verify the performance of BP estimation, the mean absolute error (MAE) and the standard deviation (SD) between the estimated BP and the auscultatory nurse measurements were calculated [9, 23, 24] as shown in Table 1. The MAE of the proposed TSPMAE algorithm was compared to that of the PMAE and MAA methods as in Table 1. In addition, the standard deviation (SD) was used to describe a measure of error variability between the auscultatory nurse measurements and the estimates obtained using the proposed method. The range of the CI (mean) of the proposed TSPMAE with the bootstrap is smaller than that of the pseudomaximum amplitude-envelope (PMAE) [12] with bootstrap for both SBP and DBP, most likely because of the decrease in the standard deviation through the increase in the pseudomeasurements using the bootstrap method for each subject as shown in Table 2. Figures 3(c) and 3(d) show that the plot of histograms has a small bias though they are roughly normal by the TSPMA using double bootstrap.

**Figure 4:**
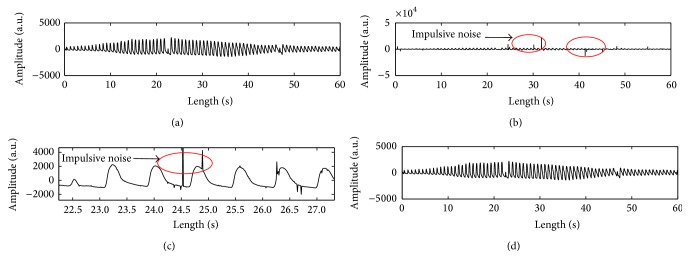
This figure shows examples as (a) oscillometric waveform (OMW); (b) OMW with impulsive noise (*γ* = 2.0); (c) enlarged figure of an OMW with impulsive noise (*γ* = 2.0); (d) cleaned OMW used the WM filter, where *γ* is a scale parameter.

**Figure 5:**
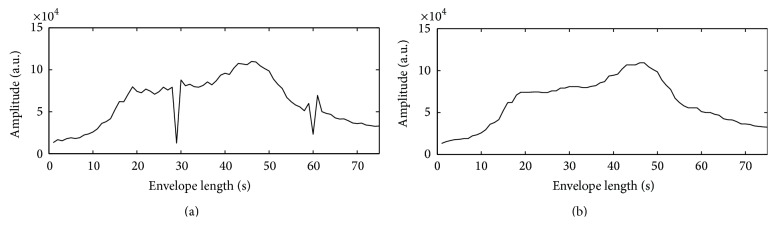
Comparison of envelops: (a) top panel, envelope, and (b) bottom panel, envelope with the WM filter.

Occasionally, the oscillometric wave signal is contaminated by additive noise such as impulsive and Gaussian noises generated from subject's moving artifact, electronic device, and environmental conditions in the processes of BP measurements. However, it is not well defined with the Gaussian model [19]. Thus, to evaluate the robustness of the TSPMAE algorithm in impulsive and Gaussian noisy environments, we generated the impulsive and Gaussian noise. First, the impulsive noise is represented by four parameters: a scale parameter *γ* > 0, an index of stability *α* ∈ (0,2], a skewness parameter *δ* ∈ [−1,1], and a location parameter *β* ∈ *R*. The scale parameter *γ* is a key factor to generate impulsive noise, which is similar to the variance of the Gaussian distribution. The stability parameter *α* = 0.5 in our paper measures the thickness of the tails of the distribution. When the skewness parameter is set to *δ* = 0 in our paper, the stable distribution is symmetric about the location parameter *β* = 0 [19]. Based on the generated impulsive noise, we presented simulation results under impulsive and Gaussian noise environments. Indeed, Figure 4 shows an example of the preprocessing of the proposed methodology using the WM filter to reduce the impulsive noise of the oscillometric wave signal with respect to one subject.  Figure 5 also shows an example of the processing of the WM filter for a noise artifact caused by subject movement.

In Table 3, we have presented the CIs of the proposed method for SBP and DBP, respectively, in impulsive noisy environments, and also compared the proposed TSPMAE with weighted median (TSPMAEWM) method with the conventional methods (MAAST and MAAGUM) in order to verify the robustness of the proposed method TSPMAEWM. Here, we can not find that the CI of the TSPMAEWM is varied due to the decrease of the *γ* from 2.0 to 0.5. In this section, we omitted the explanation of white Gaussian noise's generation because it is a basic method. The conventional MAAST and MAAGUM do not work well in white Gaussian noise contaminated environments for all SNRs. However, the proposed TSPMAEWM works well except for SNRs of 5 and 10 dB in white Gaussian noise contaminated scenarios as given in Table 4.

## 4. Discussion

The goal of this paper is to derive the improved CIs for SBP and DBP estimates when only a small number of blood pressure measurements are available. The degree of error variability between the readings obtained with the proposed method and those obtained with the auscultatory nurse method as the reference (Table 1) was investigated. The MAE of the SBP and DBP obtained through the TSPMAE is similar to that obtained with the MAA. The proposed TSPMAE method has a MAE about 5-6 mmHg with respect to the auscultatory nurse measurements. Although the proposed approach in this paper does not focus on providing robust blood pressure estimates, the result of the MAE does not fall within the 5 mmHg recommendations of the AAMI SP 10, but the result of the SD is satisfied by the AAMI [9]. In addition, we note that the TSPMAE method has also much smaller spread (i.e., small standard deviation) in the CI when compared with the MAAST, MAAGUM, and PMAE based on the average results for 85 subjects in Table 2. We also note that the SDs of the SBP and DBP of the TSPMAE method are similar to those of SBP and DBP of the MAAST as shown in columns 4 to 7 of Table 2. According to the bootstrap principle, the distributions of the SBP and DBP of the TSPMAE represent the sampling distribution of the original measurement successfully. An interesting point is that the SDs of the CI obtained from the TSPMAE method have much smaller SDs than the CI obtained from the PMAE, but the average range of the CIs for both methods is very similar. This indicates that the range of the CI fluctuates higher for the method which uses the PMAE. The range of the CI developed using the TSPMAE method is more stable across the 85 subjects. The decrease of the standard deviation in the CI results obtained by our method is clear and it demonstrates the advantage of the proposed TSPMAE method over the existing PMAE method for obtaining the CI from a small set of measurements as shown in Table 2.

The bottom histograms of Figure 3 confirm that TSPMA produces the distributions tighter than the PMA. Note that Figures 3(c) and 3(d) represent the histograms' plot along with frequency closer to normal than PMA as shown in Figures 3(a) and 3(b). Thus, we also confirmed TSPMA to overcome the weakness of small measurement of the PMA [12]. And Table 3 shows that the comparisons of CIs of the PMAE with WM (PMAEWM) and TSPMAEWM are almost unaffected in impulsive noise contaminated scenario. On the contrary, it is found that the conventional methods (MAAST and MAAGUM) represent a very weak characteristic on impulsive noise contaminated scenario. In Table 3, the PMAEWM and TSPMAEWM methods are well represented in that the robust characteristic is made regardless of the variation of the impulsive noise at contaminated scenarios from *γ* = 2.0 to *γ* = 0.5. However, the proposed TSPMAE also does not work well in low SNRs of 5 and 10 dB white Gaussian noise contaminated scenarios as given in Table 4. Unfortunately, the conventional methods (MAAST and MAAGUM) also do not work in white Gaussian noise contaminated environments from SNRs of 5 to 20 dB. In Figures 4 and 5, we present simulation results under impulsive and Gaussian noise environments. Indeed, we used the calculation of the correlation coefficient to verify the robustness of the proposed TSPMAEWM in impulsive noise contaminated oscillometric waveform. As a result, the correlation coefficient between the top panel of Figure 4(a) and the bottom panel of Figure 4(d) was 0.99, which can be considered relatively very high. In Figure 5, the measured envelope abruptly fluctuates (compared to envelopes in top and bottom panels). As a result, the measured envelope becomes smooth (compared to envelopes in top and bottom panels at envelope's length from 18 to 30 sec and from 57 to 60 sec), and all small notches in the contaminated oscillometric envelope are eliminated. Therefore, the proposed TSPMAEWM is quite effective in impulsive and Gaussian noise environments.

## 5. Conclusion

In conclusion, we demonstrated that the CI obtained using the proposed method is narrower and has a narrower standard deviation than CIs obtained using other methods. Note that this paper does not focus on accuracy directly while the accuracy in the estimates can be obtained through the standard error from the golden reference. If the standard deviation of the estimate is low and if there is no bias, then the estimates may be deemed to be accurate. The decrease of the standard deviation in the CI results is attributed to the increase in the effective number of samples due to resampling using bootstrap principles. The results indicate that the proposed methodology reduces the standard deviation and consequently improved the accuracy. Our results imply that the proposed methodology is the best available to deal with small samples of blood pressure measurements. Our proposed method outperformed conventional methods for obtaining the CI under regular recording, impulsive, and white Gaussian noisy conditions. Indeed, the proposed technique can be used extensively as a potential application for self- and home-based monitoring scenario. We expect further studies to extend this methodology for older people with stiff arteries and wide pulse pressures and we will present these results in a near future.

## Figures and Tables

**Table 1:** Summary (averaging 85 subjects with five measurements) of the MAE and SD between the auscultatory nurse measurements of TSPMAE, MAA, and PMAE.

BP (mmHg)	MAE (nurse versus MAA)	MAE (nurse versus PMAE)	MAE (nurse versus TSPMAE)	SD (nurse versus MAA)	SD (nurse versus PMAE)	SD (nurse versus TSPMAE)
SBP	6.52	6.49	6.48	5.94	5.91	5.72
DBP	5.63	5.63	5.60	5.32	5.33	5.10

**Table 2:** Comparison of average results (85 subjects with five measurements) in CIs (95%) of SBP and DBP using the MAAST, MAAGUM, PMAE, and TSPMAE, where σ is a standard deviation and L and U are lower and upper limits, respectively. MAA with ST is MAAST. MAA with the Guide to the Expression of Uncertainty in Measurement (GUM) is MAAGUM [16].

BP	SBP (σ)	DBP (σ)	SBP (σ)	SBP (σ)	DBP (σ)	DBP (σ)
(mmHg)	CI	CI	L	U	L	U
MAAST	13.5 8.1	9.3 5.7	106.7 14.3	120.2 16.5	62.4 10.4	71.7 11.0
MAAGUM	14.1 7.8	10.1 5.3	106.4 14.3	120.5 16.4	62.0 10.4	72.1 10.9
PMAE	2.6 3.1	1.5 2.3	112.4 13.9	115.0 14.9	66.7 10.5	68.2 9.9
TSPMAE	2.4 0.9	1.3 0.5	111.4 14.5	113.7 14.8	68.8 10.2	70.1 10.5

**Table 3:** Comparison of average results (85 subjects with five measurements) in CIs of SBP and DBP using the PMAEWM and TSPMAEWM in impulsive noisy environments within γ = 2.0 and γ = 0.5 where σ is a standard deviation and N/A denotes not available.

BP (mmHg)	γ	SBP (σ)	DBP (σ)	CI SBP (σ)	CI DBP (σ)
MAAST and MAAGUM	2.0	N/A	N/A	N/A	N/A
MAAST and MAAGUM	1.5	N/A	N/A	N/A	N/A
MAAST and MAAGUM	1.0	N/A	N/A	N/A	N/A
MAAST and MAAGUM	0.5	N/A	N/A	N/A	N/A
PMAEWM	2.0	114.1 14.2	67.6 10.3	2.6 3.0	1.6 2.4
PMAEWM	1.5	114.2 14.2	67.5 10.2	2.6 3.0	1.5 2.4
PMAEWM	1.0	114.2 14.3	67.5 10.2	2.6 3.0	1.5 2.3
PMAEWM	0.5	114.1 14.2	67.5 10.2	2.6 3.0	1.6 2.3
TSPMAEWM	2.0	113.5 14.1	68.6 10.3	2.5 1.0	1.4 0.6
TSPMAEWM	1.5	113.2 14.2	68.5 10.2	2.4 0.9	1.3 0.6
TSPMAEWM	1.0	113.2 14.1	68.6 10.2	2.4 0.9	1.3 0.5
TSPMAEWM	0.5	113.1 14.2	68.6 10.2	2.4 0.9	1.2 0.5

**Table 4:** Comparison of average results in CIs of SBP and DBP using the PMAEWM and TSPMAEWM under Gaussian noisy environments within SNR 5 dB to SNR 20 dB where n (= 85) is the number of subjects with five measurements and σ is a standard deviation and N/A denotes not available.

BP (mmHg)	SNR	SBP (σ)	DBP (σ)	CI SBP (σ)	CI DBP (σ)
MAAST and MAAGUM	5 dB	N/A	N/A	N/A	N/A
MAAST and MAAGUM	10 dB	N/A	N/A	N/A	N/A
MAAST and MAAGUM	15 dB	N/A	N/A	N/A	N/A
MAAST and MAAGUM	20 dB	N/A	N/A	N/A	N/A
PMAEWM	5 dB	N/A	N/A	N/A	N/A
PMAEWM	10 dB	N/A	N/A	N/A	N/A
PMAEWM	15 dB	114.3 14.8	67.4 10.1	3.5 4.4	1.7 2.5
PMAEWM	20 dB	114.1 14.1	67.4 10.2	3.1 5.3	1.5 2.1
TSPMAEWM	5 dB	N/A	N/A	N/A	N/A
TSPMAEWM	10 dB	N/A	N/A	N/A	N/A
TSPMAEWM	15 dB	113.3 14.8	68.4 10.1	3.5 1.5	1.7 0.8
TSPMAEWM	20 dB	113.1 14.1	68.4 10.2	3.1 1.4	1.5 0.7

## Abbreviations

MAA: Maximum amplitude algorithm
CP: Cuff pressure
SBP: Systolic blood pressure
DBP: Diastolic blood pressure
BP: Blood pressure
ANSI: American National Standard Institute
AAMI: Advancement of Medical Instrumental
CI: Confidence interval
ST: Student's *t*-distribution
TSPMA: Two-step pseudomaximum amplitude
PMA: Pseudomaximum amplitude
WM: Weighted median
PE: Pseudoenvelope
TSPMAE: Two-step pseudomaximum amplitude-pseudoenvelope
MA: Maximum amplitude
NPB: Nonparametric bootstrap
MCP: Mean cuff pressure
MAE: Mean absolute error
SD: Standard deviation
ME: Mean error
MAAST: Maximum amplitude algorithm with Student's *t*-distribution
MAAGUM: Maximum amplitude algorithm with Guide to the Expression of Uncertainty in Measurement
PMAE: Pseudomaximum amplitude-envelope
PMAEWM: Pseudomaximum amplitude-envelope with weighted median
TSPMAEWM: Two-step pseudomaximum amplitude-envelope with weighted median.

## References

[1] Geddes L. A., Voelz M., Combs C., Reiner D., Babbs C. F. (1982). Characterization of the oscillometric method for measuring indirect blood pressure. *Annals of Biomedical Engineering*.

[2] Drzewiecki G., Hood R., Apple H. (1994). Theory of the oscillometric maximum and the systolic and diastolic detection ratios. *Annals of Biomedical Engineering*.

[3] Ng K.-G., Small C. F. (1994). Survey of automated noninvasive blood pressure monitors. *Journal of Clinical Engineering*.

[4] Baker P. D., Westenskow D. R., Kück K. (1997). Theoretical analysis of non-invasive oscillometric maximum amplitude algorithm for estimating mean blood pressure. *Medical and Biological Engineering and Computing*.

[5] Kiers H. D., Hofstra J. M., Wetzels J. F. M. (2008). Oscillometric blood pressure measurements: differences between measured and calculated mean arterial pressure. *The Netherlands Journal of Medicine*.

[6] Pickering T. G., Hall J. E., Appel L. J. (2005). Recommendations for blood pressure measurement in humans and experimental animals. Part 1: Blood pressure measurement in humans: a statement for professionals from the subcommittee of professional and public education of the American Heart Association council on high blood pressure research. *Hypertension*.

[7] O'Brien E., Asmar R., Beilin L. (2003). European society of hypertension recommendations for conventional, ambulatory and home blood pressure measurement. *Journal of Hypertension*.

[8] Hansen S., Staber M. (2006). Oscillometric blood pressure measurement used for calibration of the arterial tonometry method contributes significantly to error. *European Journal of Anaesthesiology*.

[9] (2003). *Manual, Electronic or Automated Sphygmonanometers*.

[10] Soueidan K., Chen S., Dajani H. R., Bolic M., Groza V. (2012). Augmented blood pressure measurement through the noninvasive estimation of physiological arterial pressure variability. *Physiological Measurement*.

[11] Krakoff L. R. (2009). Confidence limits for interpretation of home blood pressure recordings. *Blood Pressure Monitoring*.

[12] Lee S., Bolic M., Groza V. Z., Dajani H. R., Rajan S. (2011). Confidence interval estimation for oscillometric blood pressure measurements using bootstrap approaches. *IEEE Transactions on Instrumentation and Measurement*.

[13] Moore D. S., Mccabe G. P. (2004). *Introduction to the Practice of Statistics*.

[14] Martin M. A. (1990). On the double bootstrap.

[15] Nankervis J. C. (2005). Computational algorithms for double bootstrap confidence intervals. *Computational Statistics & Data Analysis*.

[16] BIPM (1993). *Guide to the Expression of Uncertainty in Measurement*.

[17] Efron B., Tibshirani R. (1986). Bootstrap methods for standard errors, confidence intervals, and other measures of statistical accuracy. *Statistical Science*.

[18] Yin L., Yang R., Gabbouj M., Neuvo Y. (1996). Circuits and systems exposition: weighted median filters: a tutoral. *IEEE Transaction on Circuits and Systems II: Analog and Digital Signal Processing*.

[19] Arce G. R. (2005). *Nonlinear Signal Processing a Statistical Approach*.

[20] Chernick M. C. (2008). *Bootstrap Methods: A Guide for Practitioners and Researchers*.

[21] Zoubir A. M., Boashash B. (1998). The bootstrap and its application in signal processing. *IEEE Signal Processing Magazine*.

[22] Lee S., Jeon G., Lee G. (2013). On using maximum a Posteriori probability based on a Bayesian model for oscillometric blood pressure estimation. *Sensors*.

[23] Ahmad S., Bolic M., Dajani H., Groza V., Batkin I., Rajan S. (2010). Measurement of heart rate variability using an oscillometric blood pressure monitor. *IEEE Transactions on Instrumentation and Measurement*.

[24] Forouzanfar M., Dajani H. R., Groza V. Z., Bolic M., Rajan S. (2011). Feature-based neural network approach for oscillometric blood pressure estimation. *IEEE Transactions on Instrumentation and Measurement*.

